# Epidemiology and clinical impact of pediatric RSV co-infections after the COVID-19 pandemic: a narrative review

**DOI:** 10.3389/fped.2026.1787312

**Published:** 2026-04-24

**Authors:** Yingying Hu, Jun Li, Yixiang Zheng, Youde Cheng, Wei Li, Xu Wang, Yanqun Sun

**Affiliations:** 1 Department of Pediatrics, Nanjing Jiangbei Hospital, Nanjing, China; 2 Clinical Medical Research Center, Children's Hospital of Nanjing Medical University, Nanjing, China

**Keywords:** co-infection, disease severity, lower respiratoryTract infections, pediatrics, respiratory syncytial virus (RSV)

## Abstract

The coronavirus disease 2019 (COVID-19) pandemic profoundly disrupted the global epidemiology of respiratory syncytial virus (RSV), leading to atypical off-season surges and altering infection dynamics across different climatic zones. This review synthesizes evidence on the landscape of pediatric RSV co-infections in this transformed post-pandemic context. RSV co-infection is frequent, with human rhinovirus (HRV) being the most common viral co-pathogen and *Streptococcus pneumoniae* (Spn) and *Haemophilus influenzae* (Hi) predominating as bacterial co-infections. Critically, these co-infections are significantly associated with heightened disease severity, including more intense clinical presentations, prolonged hospitalizations, increased intensive care unit (ICU) admission rates, and greater therapeutic complexity. The relaxation of non-pharmaceutical interventions has been linked to a rebound in co-infection rates. While advances in molecular diagnostics have improved detection, and new prophylactics like nirsevimab offer promise, significant challenges remain. These include gaps in understanding pathogenic synergies, inequities in access to novel interventions, and the need for strategies to manage the ongoing evolution of RSV epidemiology. This underscores the necessity for enhanced surveillance, equitable prevention, and targeted research to mitigate the substantial burden of pediatric RSV co-infections.

## 1 Introduction

Respiratory syncytial virus (RSV) is a predominant etiological agent of lower respiratory tract infections (LRTIs) in young children worldwide, posing a substantial global health burden. In 2019 alone, RSV was estimated to be responsible for 33 million episodes of acute LRTIs, 3.6 million hospital admissions, and 101,400 in-hospital deaths among children under 5 years of age. Tragically, approximately half of these fatalities occurred in infants below six months, with a disproportionate majority concentrated in developing regions (1). This vulnerability is underscored by the fact that nearly all children experience at least one RSV infection by the age of two, highlighting its ubiquitous nature (2).

The COVID-19 pandemic unexpectedly altered the traditional seasonality and epidemiology of RSV. Public health interventions implemented to curb SARS-CoV-2 transmission, such as masking and social distancing, initially led to a marked suppression of RSV circulation (3). However, the subsequent easing of these non-pharmacological interventions (NPIs) was associated with a dramatic and out-of-season resurgence of RSV infections. This surge overwhelmed pediatric healthcare systems in many countries with unprecedented caseloads of acute respiratory illnesses, revealing a susceptible pediatric population with an immunity debt (4–7).

While the pathogenesis of RSV as a sole pathogen is well-studied, a critical and increasingly recognized aspect of its clinical presentation is co-infection with other respiratory pathogens. Co-infections with viruses like Human rhinovirus (HRV) or bacteria such as *Streptococcus pneumoniae* (Spn) may significantly modify the disease course, potentially leading to increased severity, complications, and prolonged hospitalization. Therefore, this article aims to systematically review the current landscape of pediatric RSV co-infections. We will synthesize evidence on the spectrum of co-circulating pathogens, their epidemiological characteristics, impact on clinical severity, and advances in diagnostic approaches. Finally, we will discuss contemporary therapeutic and preventive strategies, with the objective of providing a comprehensive reference to inform clinical decision-making and guide future research directions.

## 2 Etiologic spectrum of pediatric RSV co-infections

**Figure 1:**
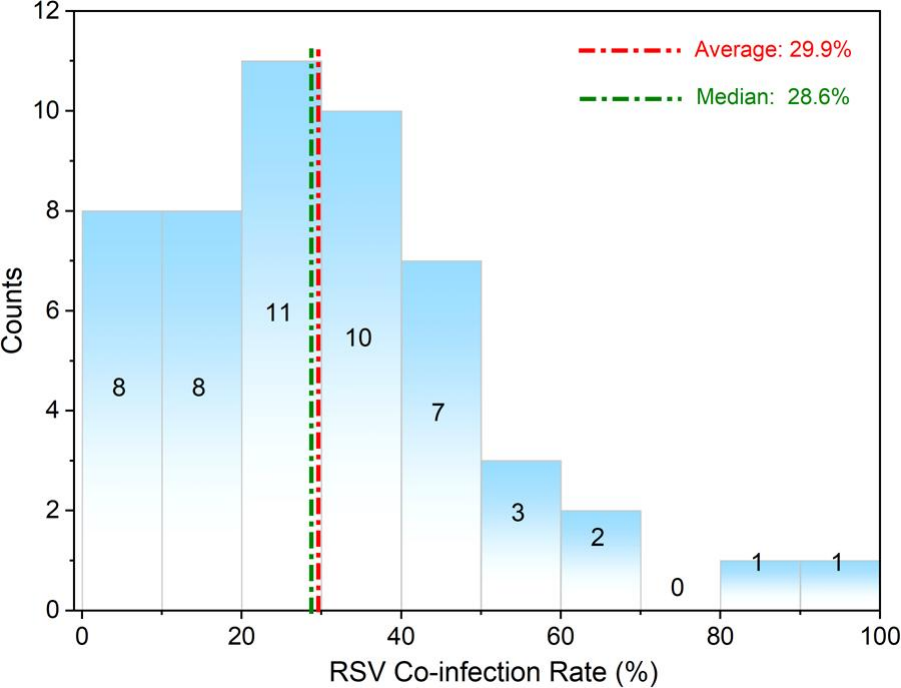
The distribution map of RSV co-infection rates reported in the literature.

RSV co-infection is prevalent among patients with acute respiratory infections. Our analysis indicated an overall co-infection rate of 29.9% (Average value) and 28.6% (Median value) (Figure 1). The landscape of pathogens involved in pediatric RSV co-infections is diverse, encompassing viruses, bacteria, and atypical organisms such as Mycoplasma. Different pathogen combinations exert distinct effects on disease progression and clinical outcomes. Post-pandemic molecular surveillance has revealed a shift toward increasingly complex polymicrobial profiles, with co-detection rates rising by 10%–15% among hospitalized children. This trend underscores the necessity of targeted etiological profiling to guide appropriate clinical management (8).

The COVID-19 pandemic has significantly impacted the epidemiology of respiratory infections, including RSV. Post-pandemic, many regions have observed a marked increase in the incidence and severity of RSV infections, largely attributed to the concept of “immunity debt"—a result of reduced exposure to respiratory pathogens during the pandemic due to NPIs. For instance, in Malaysia, the RSV positivity rate sharply increased to 36.3% in 2022, which was higher than pre-COVID levels. (9) Similarly, in Taiwan, the positive infection rate of RSV reached 33.6% in 2022, which was much higher than the level in 2018–2019 (16.7%). (10) Additionally, the incidence of co-infections has also surged. In a cohort study by Stobbelaar et al., children with RSV infections who were co-infected with other respiratory viruses had a significantly longer hospital stay, with a median duration of 8 days, compared to those with RSV alone. These patients also had higher rates of admission to the pediatric intensive care unit (PICU). (11) Co-infections with Spn were also found to increase the likelihood of severe RSV-related hospitalizations, further compounding the impact on public health. (12)

Moreover, the severity of RSV infections has also been exacerbated. In a study conducted in Yunnan, China, patients in the post-COVID period exhibited significantly higher levels of inflammatory markers such as C-reactive protein (CRP) and Interleukin 6 (IL-6), with a concomitant rise in severe LRTIs and mechanical ventilation rates. This increase in severity was particularly notable among children and immunocompromised adults (13).

These findings underscore the heightened clinical burden of RSV in the post-COVID era, emphasizing the importance of monitoring RSV activity and the need for targeted prevention and treatment strategies, particularly in vulnerable populations.

### 2.1 Virus–virus co-infections

**Figure 2:**
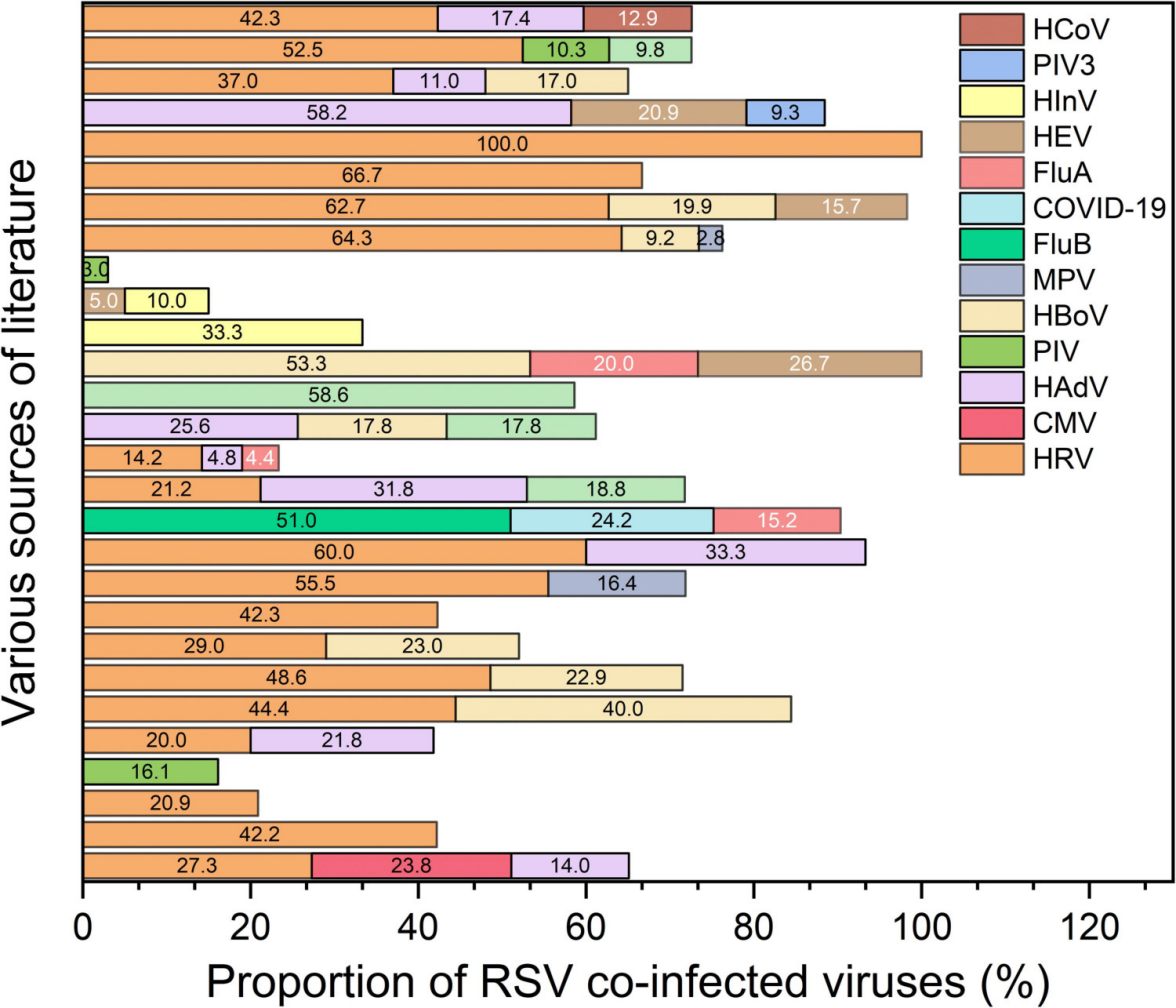
The proportions of RSV co-infection with various viruses as reported in different literature. HBoV: Human Bocavirus. PIV3: Parainfluenza Virus 3. HInV: Human Influenza Virus. HEV: Human Enterovirus. Flu A: Influenza A Virus. COVID-19: Coronavirus Disease 2019. Flu B: Influenza B Virus. MPV: Metapneumovirus. HCoV: Human Coronavirus. HAdV: Human Adenovirus. CMV: Cytomegalovirus. HRV: Human Rhinovirus.

We find that HRV was the most frequently identified virus in cases of viral co-infection associated with RSV (Figure 2). HRV remains the most prevalent viral co-pathogen, identified in 20%–48% of RSV-positive pediatric hospitalizations across Asian and European cohorts (11, 14–18). Prospective studies from Vietnam and Spain have demonstrated a synergistic interaction between RSV and HRV, which amplifies airway hyper-responsiveness through shared activation of Toll-like receptor 3 (TLR3) and upregulation of Interleukin-8 (IL-8). This synergy is clinically associated with prolonged wheezing episodes—extending the duration by a mean of 2.1 days—and a 2.5-fold increased risk of asthma development at 5-year follow-up (14, 19). In subtropical regions such as Japan, multiplex Polymerase Chain Reaction (PCR) surveillance conducted between 2020 and 2022 detected HRV–RSV co-infections in 22% of cases, often presenting as a biphasic illness characterized by initial rhinorrhea progressing to lower respiratory tract involvement (8).

Human adenovirus (HAdV) is a relatively common RSV coexisting virus, accounting for generally around 20% to 50%, while Metapneumovirus (MPV) is not common. Human bocavirus (HBoV), detected in 10%–15% of East Asian cases, exacerbates RSV-induced cytokine storms through its persistent, non-enveloped replication in airway epithelial cells. This co-infection is frequently associated with mixed lobar and interstitial pulmonary infiltrates and elevated lactate dehydrogenase levels (20). Retrospective analyses from South Korea further correlate HBoV co-infection with refractory clinical courses, showing an odds ratio (OR) of 1.8 for prolonged fever lasting more than 7 days (21).

Although less common (5%–10%), co-infections involving Parainfluenza virus (PIV) or Influenza virus (IV) can potentiate hypoxemia via neuraminidase-mediated mucus hypersecretion. Studies from Qatar and Malaysia indicate that these co-infections lead to a 15% increase in the need for supplemental oxygen, particularly among unvaccinated children (9, 22). Meta-analyses confirm notable regional variations in viral co-detection patterns, such as the predominance of HRV in temperate climates (OR 1.9) vs. PIV in subtropical regions (OR 2.2), influenced by humidity-driven transmission dynamics (23, 24).

### 2.2 Viral–bacterial co-infections

Viral–bacterial co-infections are frequently observed in children with RSV and are associated with more severe clinical outcomes. A study from Portugal reported two off-season RSV epidemics (June 2021–February 2022 and May–October 2022), identifying Hi and Spn as the most common bacterial co-pathogens. Children co-infected with potentially pathogenic bacteria had significantly higher rates of ICU admission (29.7% vs. 3.5%, *p* < 0.001), longer median hospitalization durations (7 days vs. 5 days, *p* < 0.001), and a 13-fold increased risk of severe disease (RR: 13.2, 95% CI: 7.3–23.9) (25).

**Figure 3:**
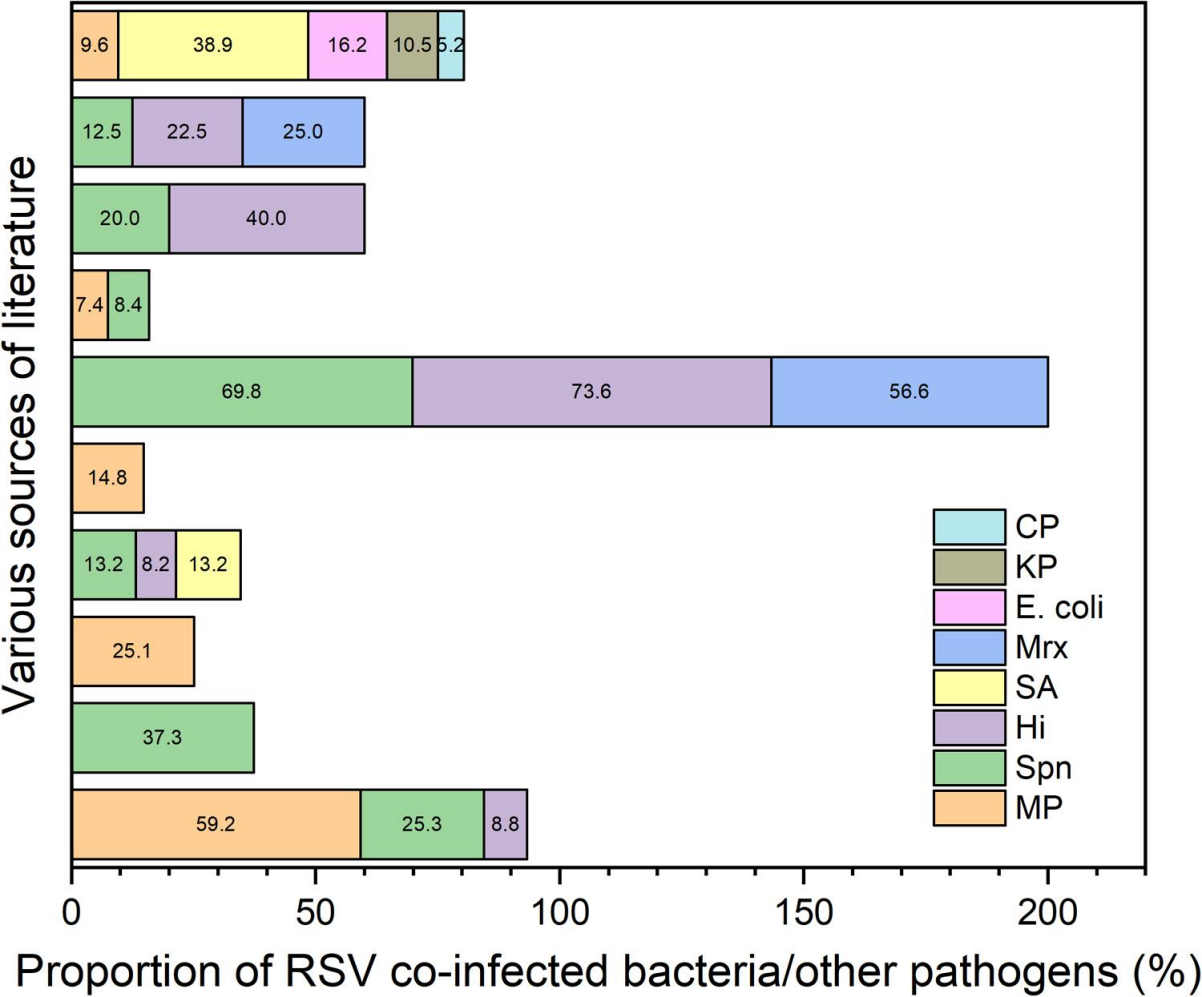
The proportions of RSV co-infection with various bacteria or pathogens as reported in different literature. CP: Chlamydia Pneumoniae. KP: Klebsiella Pneumoniae. E. coli: Escherichia Coli. Mrx: Moraxella Catarrhalis. SA: Staphylococcus Aureus. Hi: Haemophilus influenzae. Spn: Streptococcus pneumoniae. MP: Mycoplasma Pneumoniae.

Spn is the predominant bacterial pathogen in RSV-associated viral–bacterial co-infections (Figure 3). A systematic review by Besteman et al. highlighted extensive biological and clinical interactions between RSV and Spn in the pathogenesis of pediatric respiratory infections. RSV infection disrupts the respiratory epithelial barrier and upregulates adhesion molecules such as platelet-activating factor receptor and intercellular adhesion molecule-1, enhancing the colonization and invasive potential of Spn and thereby exacerbating pulmonary infection severity (12). A 2020 study by Do et al. in Vietnam further confirmed that children co-infected with RSV and Spn exhibited significantly elevated procalcitonin levels and were more likely to develop severe complications such as respiratory failure (26).

*Haemophilus influenzae* (Hi) is another frequently identified bacterial co-pathogen. In a study on community-acquired pneumonia among hospitalized children in Suzhou, China, Jiang et al. reported that RSV and Hi co-infections accounted for 38.3% of all RSV–bacterial co-infection cases. These children demonstrated higher C-reactive protein levels and prolonged hospital stays compared to those with RSV monoinfection (15). Additionally, pathogens such as *Staphylococcus aureus* (SA) and *Moraxella catarrhalis* have also been documented in RSV co-infections. A 2022 study by Lin et al. indicated that RSV and SA co-infection significantly increased the risk of complications such as empyema, suggesting a poorer clinical prognosis (27). A Chinese study from Suzhou focused on RSV-positive newborns, with particular emphasis on bacterial co-infections. It found that Staphylococcus aureus was the most frequently identified bacteria. This co-infection was further correlated with a higher risk of death in this patient population (28).

### 2.3 Viral–mycoplasma co-infections

*Mycoplasma pneumoniae* (MP) is the primary atypical pathogen involved in RSV–mycoplasma co-infections. In a study on acute lower respiratory tract infections among hospitalized children in Guangxi, China, Fu et al. found that RSV and MP co-infections constituted 12.8% of all RSV co-infection cases. These patients often presented with complex clinical features, including persistent high fever and prolonged pulmonary rales, in addition to typical RSV symptoms such as wheezing and cough. Laboratory findings indicated normal or mildly elevated white blood cell counts, alongside increased levels of C-reactive protein and procalcitonin (29). Further supporting these results, Jiang et al. reported that co-infected children often exhibited mixed radiographic patterns, including bronchopneumonia and interstitial pneumonia. Effective management generally requires combination therapy with antiviral agents and macrolide antibiotics, typically involving extended treatment durations (15).

## 3 Epidemiological characteristics of pediatric RSV co-infections

### 3.1 Age distribution

**Figure 4:**
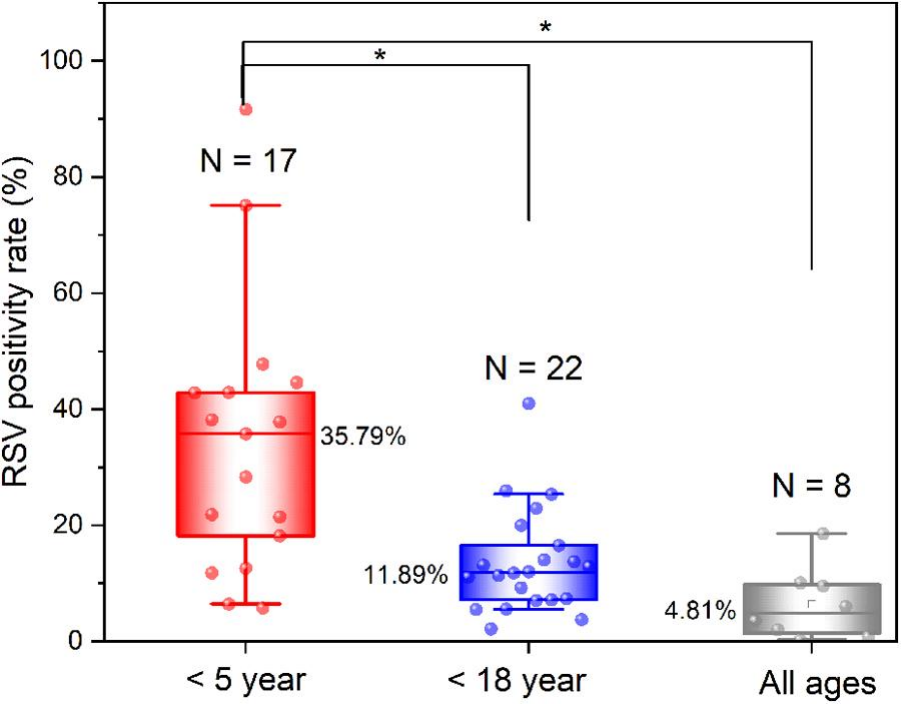
Box plot of RSV infection rates in patients with acute respiratory tract infections reported in the literature, by different age groups. N: the number of documents. *: *p* < 0.05. The percentages marked in the figure represents the median RSV positive value.

The incidence of RSV co-infection in children demonstrates a clear correlation with age, exhibiting distinct patterns across different pediatric groups. We find a significantly increased risk of RSV co-infection in children younger than five years, with the risk being most pronounced among infants under six months of age (Figure 4). In a study conducted in Eastern China between 2009 and 2013, Cui et al. reported that 89.1% of children with RSV infection were under five years old, of whom 43.3% were under one year of age. Within this youngest cohort, the co-infection rate reached 37.3% (20). A 2021 study conducted in Hefei, China, demonstrated that younger patients, particularly those under 2 years of age, exhibited a higher incidence of positive cases (30). Similarly, Do et al. observed in a Vietnamese cohort that 31% of children under two years old had RSV co-infections, with the risk inversely correlated with age (19). This pattern is attributed to the immaturity of the infant immune system and underdeveloped respiratory mucosal barrier function. As children grow older and acquire more robust immunity, the incidence of RSV co-infection declines. Although Fu et al. noted a relatively higher proportion of RSV and MP co-infections in children over five years old, the overall co-infection rate in this age group remains significantly lower than that in infants and toddlers (29). Before the COVID-19 pandemic, RSV infections primarily occurred in children under two years of age; however, the pandemic altered the age distribution of RSV infections. A study by Zhou et al. indicated a significant increase in RSV infection rates among the 3–6 years age group in the post-pandemic period (31).

The phenomenon of “immunity debt” resulting from reduced pathogen exposure during strict NPI periods likely contributed to the size of the susceptible pediatric population, fueling these atypical outbreaks. Maternal antibody and prior RSV exposure may also have shaped this altered susceptibility profile. Most infants are born with transplacentally acquired maternal antibodies against RSV, but this protection wanes quickly (5). In a neonatal cohort with acute lower respiratory tract infection, RSV positivity was 11.79%, and infection became more prevalent after the first week of life, likely owing to increasing environmental exposure together with declining maternally derived transplacental antibodies (28). In addition, RSV infection induces only partial immunity, allowing reinfections throughout life (5), while prior infection may shorten viral shedding even if it does not completely prevent reinfection (32). Consistent with reduced opportunities for prior exposure during the COVID-19 period, RSV-specific antibody levels in children declined in the post-pandemic era, and the age at infection shifted upward, with one study reporting a median age of 16.4 months compared with 8.1 months before the pandemic (13, 33).

### 3.2 Seasonal distribution

The COVID-19 pandemic profoundly disrupted the global seasonal epidemiology of RSV, though the nature and timing of these disruptions varied considerably across geographical regions and climate zones. A common pattern emerged, characterized by delayed, off-season outbreaks, increased intensity, and an upward shift in the age of infection. In temperate zones, RSV activity nearly vanished during the initial pandemic period (2020–2021 season), followed by historically anomalous resurgences. For instance, in the United States, the 2021–22 season began 21 weeks earlier (May) and peaked in July (34). Similarly, Hubei, China, experienced an unprecedented summer outbreak in 2021. After the discontinuation of NPIs in December 2022, RSV activity in Hubei peaked unusually late in April 2023 (positivity rate 50.6%), and only partially returned to its traditional seasonal pattern by 2024 (31). Australia witnessed a large-scale off-season spring-summer outbreak from late 2020 to early 2021, starting in September and peaking in December (positivity rate 37%–48%) (35). Changes in subtropical regions were more complex. Northern Taiwan saw a complete absence of an RSV epidemic season in 2021, with resurgence delayed by 2–3 months in 2022–2023. This period was also marked by an increase in the mean age of infected children from 16.6 to 26.1 months, and a doubling to 52% in the proportion of cases among children aged 2–5 years (10). In southwestern Saudi Arabia, the peak shifted to September 2022, a significant deviation from the traditional winter peak (36), while in Israel, an unusual spring outbreak in 2021 was linked to the relaxation of NPIs and school reopenings (37, 38). Tropical regions experienced missed or extremely delayed epidemic seasons. In Brazil, no RSV was detected from May to August 2020, resulting in the loss of a full season—a delay of approximately 88 weeks (37). In South Africa, detection rates fell by 98% in 2020; however, the subsequent 2020–2021 season featured two consecutive high-activity periods, extending the total epidemic duration to 30 weeks, far exceeding the historical average of 17 weeks (37). Japan presented an intra-country contrast: hospitalizations for RSV dropped to zero in Hokkaido during the NPI period (38), whereas Tokyo reported a large-scale outbreak in the spring of 2021 (37). Among the drivers of these changes, non-pharmaceutical interventions were the most critical. School closures were significantly associated with reduced RSV activity (bias increase 1.31%, *p* = 0.04), and their reopening consistently triggered resurgence (37). Globally, RSV activity is estimated to have decreased by 85%–99% during the height of restrictions (38). Pediatric RSV co-infections generally follow the epidemiological patterns of sole RSV infections, though their specific timing is influenced by regional climate. In temperate regions, both RSV infections and co-infections predominantly occur in winter and spring (Supplementary Table A1). A study in Eastern China identified peak RSV activity in autumn and winter, with over 65% of co-infections occurring between October and February (20), a pattern mirrored in Qatar where RSV and co-infections were significantly more prevalent during winter months (22). Research in Taiwan, China (2018–2023) showed the RSV epidemic season in the north occurring from August to January, a shift from the previously reported bimodal distribution (peaks in March–May and August–October) observed over prior decades—a change potentially attributed to climatic variations and the disruptive impact of pandemic-era infection control measures (39, 40).

In tropical and subtropical regions, RSV and associated co-infections circulate year-round, often with a modest increase during rainy seasons. In Vietnam, co-infection rates were approximately 15% higher during the rainy season (May–October) compared to the dry season (19). Recent post-pandemic surveillance (2020–2023) in eastern China documented a significant 15% rebound in co-infection rates, associated with the relaxation of NPIs and off-season RSV resurgences, particularly during summer months (41). Furthermore, the seasonal patterns of specific co-infecting pathogens vary. For example, RSV and IV co-infections typically peak during the influenza season in winter, whereas RSV and Spn co-infections are most frequent during the RSV season in winter and spring (12).

### 3.3 Geographic variation

The epidemiology of pediatric RSV co-infections exhibits considerable geographic heterogeneity, influenced by a range of regional factors including climatic conditions, population density, sanitation levels, and healthcare infrastructure. In developing countries or regions, factors such as limited economic resources, inadequate public health infrastructure, and high population density contribute to both a higher incidence of RSV co-infections and increased disease severity. Studies by Do et al. in Vietnam reported co-infection rates exceeding 30%, with severe cases accounting for 18.2% of these co-infected individuals (19, 26).

In contrast, developed regions generally experience lower rates of RSV co-infection and milder clinical outcomes, attributable to better sanitary conditions, advanced medical resources, and effective public health interventions. For instance, a study by Janahi et al. in Qatar documented a co-infection rate of 33%, yet the proportion of severe cases was notably lower, at only 7.4% (22).

Furthermore, the distribution of co-infecting pathogens varies geographically. In Eastern China, co-infections involving RSV and HRV are frequently observed (20), whereas in Vietnam, RSV and Streptococcus pneumoniae co-infections represent a more prominent pattern (26).

## 4 Impact of RSV co-infections on disease severity in children

### 4.1 Exacerbation of clinical symptoms

RSV co-infections are generally associated with a greater clinical burden in children than RSV monoinfection. Across studies, co-infected children more frequently presented with prolonged fever, severe cough, wheezing, respiratory distress, and, in some cases, systemic manifestations such as vomiting, diarrhea, lethargy, and somnolence (Table 1, Supplementary Table A1). Do et al. reported that RSV–HRV co-infection prolonged fever by 1.5–2 days and increased the frequency of cough and wheezing, particularly at night, thereby substantially impairing sleep quality (19). In the same study, RSV–bacterial co-infection was associated with dyspnea in 81.8% of cases, and some children progressed to respiratory failure requiring ventilatory support (26). Cui et al. further showed that diarrhea was more common in co-infected children than in those with RSV monoinfection (18.2% vs. 12.3%) (20). Overall, RSV co-infection significantly increases the risk of severe illness and the need for critical care (31).

**Table 1:** Coinfection rates and comparative clinical manifestations/morbidity-mortality impact by pathogen group.

Reference	Groups	Sample, N	Proportion within study cohort	Fever prevalence	Length of stay (days)	Clinical manifestations/indicators	Severe disease/severity indicators	Symptomatology and clinical-course comparison between coinfection and monoinfection
Do et al., 2016. (19)	Single RSV infection	201	31.8%	56% (>37.5 °C); 23% (≥38.5 °C)	Median 6 (IQR 5–7); ≥7: 38%	Tachypnea: 83%; Chest indrawing: 96%; Wheeze: 96%; Rhinorrhea: 91%; Low SpO2: 14%; Cyanosis: 3%	Severe disease: 18%; median clinical score: 6 (4–7)	NA
	Single RV infection	87	13.8%	57% (>37.5 °C); 15% (≥38.5 °C)	Median 6 (IQR 5–8); ≥7: 49%	Tachypnea: 86%; Chest indrawing: 94%; Wheeze: 88%; Rhinorrhea: 81%; Low SpO2: 10%; Cyanosis: 4%	Severe disease: 25%; median clinical score: 5 (4–7)	
	Single MPV infection	17	2.7%	41% (>37.5 °C); 29% (≥38.5 °C)	Median 6 (IQR 4–7);≥7: 41%	Tachypnea: 82%; Chest indrawing: 88%; Wheeze: 94%; Rhinorrhea: 88%; Low SpO2: 7%; Cyanosis: 0%	Severe disease: 18%; median clinical score: 5 (4–6)	
	Single PIV-3 infection	18	2.8%	33% (>37.5 °C); 11% (≥38.5 °C)	Median 6 (IQR 5–7); ≥7: 39%	Tachypnea: 78%; Chest indrawing: 83%; Wheeze: 94%; Rhinorrhea: 94%; Low SpO2: 6%; Cyanosis: 0%	Severe disease: 17%; median clinical score 4.5 (4–6)	
	RSV- coinfection	101	16.0%	NR	NR	NR	NR	
	Any viral coinfection	199	31.5%	NR	NR	NR	NR	
Janahi et al., 2017. (22)	RSV	127	34.4%	74.40%	≥4: 82.4%	Cough: 100%; Wheeze: 68%; Crackles/crepitations: 78%; Chest retractions: 77%; Apnea: 7.3%	ICS grade: 3–4: 57.6%; assisted ventilation: 10.0%	Coinfection groups showed some higher symptom frequencies (e.g., wheeze, crackles, chest retractions, apnea), but these did not translate into a consistent increase in burden indicators.
	RV	36	9.8%	71.40%	≥4: 91.7%	Cough: 97.2%; Wheeze: 72.2%; Crackles/crepitations: 85.7%; Chest retractions: 82.9%; Apnea: 3.1%	ICS grade: 3–4: 65.7%; assisted ventilation: 16.7%	
	RSV+RV	25	6.8%	79.20%	≥4: 87.5%	Cough: 100%; Wheeze: 83.3%; Crackles/crepitations: 91.7%; Chest retractions: 75%; Apnea: 13%	ICS grade: 3–4:58.3%; assisted ventilation: 4.3%	
	RSV+any other non-RV	33	8.9%	87.50%	≥4:90.9%	Cough: 100%; Wheeze: 68.8%; Crackles/crepitations: 96.2%; Chest retractions: 90.3%; Apnea: 4.2%	ICS grade: 3–4: 56.7%; assisted ventilation: 6.1%	
	RV + any other non-RSV	22	6.0%	80.00%	≥4:86.4%	Cough: 100%; Wheeze: 59.1%; Crackles/crepitations: 76.2%; Chest retractions: 59.1%; Apnea: 5.3%	ICS grade: 3–4:45.5%; assisted ventilation: 9.5%	
	Others	72	19.5%	79.70%	≥4: 84.8%	Cough: 97.1%; Wheeze: 62.7%; Crackles/crepitations: 93.3%; Chest retractions: 82%; Apnea: 13.8%	ICS grade: 3–4: 51.5%; assisted ventilation: 12.3%	
Nguyen et al., 2020. (26)	RSV alone	59	84.3%	37.30%	NR	Cough: 97.1%; Wheeze: 62.7%; Crackles/crepitations: 93.3%; Chest retractions: 82%; Apnea: 13.8%	All enrolled patients had severe RSV pneumonia; in-hospital mortality: 0%	All participants had severe RSV pneumonia, but in-hospital mortality occurred only in the bacterial coinfection group (18.2% vs. 0%). Strong evidence that bacterial coinfection increased fever burden and laboratory inflammation.
	RSV+bacterial coinfection	11	15.7%	81.80%	NR	Wheeze: 81.8%; Rhinorrhea: 27.3%; Diarrhea: 18.2%; Tachycardia: 54.6%	All enrolled patients had severe RSV pneumonia; in-hospital mortality: 18.2%	
Asseri et al., 2025. (36)	RSV	230	54.4%	94.80%	Median 7 (IQR 4–10)	Wheeze: 71.3%; Cough: 95.2%; Rhinorrhea: 87.8%; Dyspnea: 95.7%; Feeding intolerance: 89.6%	PICU/HFNC: 35.7%; mechanical ventilation: 5.2%; death: 1.3%	RSV had higher rates of wheeze, cough, rhinorrhea, dyspnea, and feeding intolerance than several comparator viruses, indicating a heavier clinical manifestation profile despite the lack of coinfection categories.
	SARS-CoV-2	69	16.3%	95.70%	Median 5 (IQR 3–10)	Wheeze: 68.1%; Cough: 81.2%; Rhinorrhea: 84.1%; Dyspnea: 76.8%; Feeding intolerance: 78.3%	PICU/HFNC: 20.3%; mechanical ventilation: 1.4%; death: 1.4%	
	Influenza A	79	18.7%	100%	Median 5 (IQR 3–7)	Wheeze: 58.2%; Cough: 81.0%; Rhinorrhea: 77.2%; Dyspnea: 68.4%; Feeding intolerance: 67.1%	PICU/HFNC: 10.1%; mechanical ventilation: 2.5%; death: 0%	
	Influenza B	45	10.6%	100%	Median 4 (IQR 3–11.5)	Wheeze: 46.7%; Cough: 93.3%; Rhinorrhea: 71.1%; Dyspnea: 68.9%; Feeding intolerance: 44.4%	PICU/HFNC: 24.4%; mechanical ventilation: 6.7%; death: 2.2%; PICU Hospital stay 9 days(2–68)	
Choo et al., 2022. (21)	MP non-coinfection	82	56.6%	98.80%	5.7 ± 3.8	NR	ICU: 0%; oxygen therapy: 7.3%; Treatment response: good 46.3%; slow response 46.3%; poor response 7.3%	Coinfection was associated with worse morbidity: longer fever duration, longer hospital stay, poorer treatment response, and greater odds of severe pneumonia (aOR 4.60). ICU admission remained 0% in both groups; oxygen therapy was similar (4.8% vs. 7.3%).
	MP + respiratory virus coinfection	63	43.4%	100%	7.7 ± 4.7	NR	ICU: 0%; oxygen therapy: 4.8%; associated with severe pneumonia (aOR 4.60); Treatment response was poorer: good 25.8%; slow response 56.5%; poor response 17.7%	
Jiang et al., 2017. (15)	Single virus	118	19.9%	35.60%	NR	Wheezing: 50.8%; Dyspnea: 9%	oxygen requirement: 11.4%; PICU: 0%	Mixed infections did not show a uniformly worse burden than monoinfection. Oxygen requirement was 11.1% in mixed viruses and 9.5% in mixed bacteria/viruses vs. 11.4% in single-virus infection; PICU admission was 7.4% in mixed viruses, 3.5% in mixed bacteria/viruses, and 0% in single-virus infection.
	Single bacteria	182	30.7%	67.50%	NR	Wheezing: 32.4%; Dyspnea: 7.9%	oxygen requirement: 2.7%; PICU: 0.6%	
	Mixed viruses	28	4.7%	51.80%	NR	Wheezing: 51.9%; Dyspnea: 7.7%	oxygen requirement: 11.1%; PICU: 7.4%	
	Mixed bacteria/viruses	209	35.2%	53.70%	NR	Wheezing: 47.8%; Dyspnea: 8.5%	oxygen requirement: 9.5%; PICU: 3.5%	
	Mixed bacteria	56	9.4%	57.30%	NR	Wheezing: 44.1%; Dyspnea: 8.1%	oxygen requirement: 8.1%; PICU: 2.2%	
Garcia-Garcia et al., 2017. (14),	Single infection	192	78.7%	47.4% (>37.9 °C)	2.6 ± 1.8	Abnormal chest x-ray: 38%; Antibiotic use: 13.5%	NR	Coinfection was associated with worse major outcomes: higher duration and proportion of fever (coinfection vs. single infection)
	Viral coinfection	52	21.3%	57.7% (>37.9 °C)	3.1 ± 2.0	Abnormal chest x-ray: 34%; Antibiotic use: 19%	NR	

HFNC, High-flow nasal cannula; ICS, Index clinical score; LOS, Length of stay; MP, *Mycoplasma pneumoniae*; NR, Not reported; PICU, Pediatric intensive care unit; RSV, Respiratory Syncytial Virus; RV, Rhinovirus; IQR, Interquartile range; NA, Not availab.

As shown in Table 1, coinfection was frequent across studies and was consistently associated with worse clinical-course indicators than monoinfection. Reported frequencies included 16.0% for RSV coinfection and 31.5% for any viral coinfection in Do et al. (19); 6.8% for RSV + RV and 8.9% for RSV+non-RV coinfection in Janahi et al. (22); 15.7% for RSV+bacterial coinfection in Nguyen et al. (26); and 21.3% for viral coinfection in Garcia-Garcia et al. (14) Direct comparisons suggested that coinfection was associated with a higher fever burden, more severe respiratory manifestations, and longer hospitalization. For example, Garcia-Garcia et al. reported higher fever prevalence (57.7% vs. 47.4%) and longer hospital stay (3.1 ± 2.0 vs. 2.6 ± 1.8 days) in children with viral coinfection (14), while Choo et al. found that MP + respiratory virus coinfection was associated with longer hospitalization (7.7 ± 4.7 vs. 5.7 ± 3.8 days), poorer treatment response, and more severe pneumonia (Table 1) (21)

Febrile episodes were documented in both RSV and non-RSV infections, but the overall pattern suggests a heavier fever burden in coinfection. In Do et al., fever >37.5 °C occurred at similar rates in single RSV and RV infections (56% vs. 57%), although high fever (≥38.5 °C) was more frequent in RSV (23% vs. 15%) (19). However, disease severity was not uniformly highest in RSV monoinfection. By contrast, studies directly comparing mono-infection and co-infection groups showed a clearer difference. In Janahi et al., fever prevalence increased from 74.4% in RSV monoinfection to 79.2% in RSV + RV and 87.5% in RSV+non-RV coinfection, with numerically higher frequencies of wheeze, crackles/crepitations, and chest retractions in the coinfection groups (22). Likewise, Nguyen et al. showed that RSV+bacterial coinfection was associated with substantially higher fever prevalence than RSV monoinfection (81.8% vs. 37.3%), and in-hospital mortality occurred only in the coinfection group (18.2% vs. 0%) (Table 1) (26)

Taken together, the available evidence indicates that RSV co-infection, whether viral or bacterial, is associated with more severe symptoms, longer hospitalization, poorer clinical outcomes, and, in some studies, higher mortality than RSV monoinfection (19, 20, 26, 31).

### 4.2 Prolonged hospitalization

Co-infection with RSV is a significant predictor of extended hospital stays. Multiple studies have confirmed that the length of hospitalization is markedly longer in co-infected children compared to those with single RSV infections. In a study from Suzhou, China, Jiang et al. reported a mean hospitalization duration of 5.2 days for children with sole RSV infection, which increased to 7.8 days in cases of RSV co-infection (15). Similarly, Do et al. observed that children with RSV–HRV co-infection were hospitalized for an average of 6.5 days—1.3 days longer than those infected with RSV alone (19). Janahi et al. further corroborated these findings in a Qatari cohort, reporting median hospitalization durations of 7 days for co-infected children vs. 5 days for those with monoinfection (22). Prolonged hospitalization not only increases healthcare resource utilization but also imposes substantial economic and psychological burdens on families.

### 4.3 Increased risk of complications

RSV co-infections are linked to a higher incidence of complications, including pneumonia, respiratory failure, myocarditis, and encephalitis. According to a review by Besteman et al., children co-infected with RSV and Streptococcus pneumoniae are at significantly elevated risk of developing pneumonia, including severe forms complicated by lung abscesses or empyema (12). Do et al. also reported respiratory failure in 18.2% of children with RSV–bacterial co-infection, a rate substantially higher than the 5.7% observed in those with single RSV infections (26). Furthermore, co-infections may contribute to long-term sequelae such as airway remodeling and bronchiectasis, adversely affecting respiratory development and long-term health. Garcia-Garcia et al. demonstrated that children with RSV–HRV co-infection had a 2.5-fold higher risk of developing asthma by 6–8 years of age compared to those with RSV infection alone (14). A study conducted in Tokyo, Japan, demonstrated that co-infection of RSV with coronavirus (OR = 7.282, 95% CI: 2.289–25.95), parainfluenza virus (OR = 5.826, 95% CI: 2.377–13.78), adenovirus (OR = 5.097, 95% CI: 1.965–13.36), or HRV (OR = 4.037, 95% CI: 1.908–8.338) was significantly associated with exacerbation of bronchial asthma (BA) when compared to RSV monoinfection (42).

Immunocompromised patients, including children with malignancies undergoing chemotherapy, are at significantly higher risk for severe RSV infections due to their weakened immune systems. A study conducted in Suzhou, China, revealed that neonates with underlying conditions, including malignancies, had a higher risk of severe RSV infections, with 21.8% of RSV cases categorized as severe (28). Additionally, a study in Qatar, which analyzed viral infections in children hospitalized with acute bronchiolitis, found that RSV was the leading cause, and although not specific to immunocompromised children, it highlighted that severe RSV cases were more frequent in younger children under 6 months of age, a critical group for those with underlying health conditions (22). Co-infections with RSV and other respiratory pathogens, such as HAdV, HRV, and MPV, were shown to worsen the clinical outcomes in children with underlying conditions, including those undergoing chemotherapy (40, 42). Furthermore, the use of palivizumab, a monoclonal antibody, has been proven to significantly reduce RSV hospitalizations in high-risk populations, with studies showing an 88.4% efficacy in preventing RSV-related hospitalizations among infants (43).

### 4.4 Increased treatment complexity

The management of RSV co-infections is notably more challenging due to the involvement of multiple pathogens, necessitating combination therapies tailored to pathogen-specific susceptibility and resistance profiles. For instance, children with RSV–bacterial co-infection require both antiviral and antibiotic agents. However, antibiotic selection must be guided by culture and sensitivity testing; inappropriate choices may compromise therapeutic efficacy and promote antimicrobial resistance (26). In cases of RSV–MP co-infection, a regimen combining antiviral and macrolide antibiotics is indicated, though treatment failure may occur due to macrolide-resistant strains (29). Co-infected children often exhibit slower response to therapy, requiring close clinical monitoring and timely adjustment of treatment strategies. Lin et al. reported in 2022 that the efficacy of antibiotic therapy in children with RSV–bacterial co-infection was 72.3%, lower than the 85.6% observed in those with pure bacterial infections (27).

## 5 Diagnostic approaches for pediatric RSV co-infections

### 5.1 Conventional diagnostic methods

Viral Culture: A classical method for RSV detection, has limited utility in diagnosing co-infections. It is labor-intensive, requires prolonged incubation (5–7 days), and exhibits low sensitivity, especially later in infection or post-treatment, leading to a substantial decline in positivity rate. Furthermore, it is unsuitable for the simultaneous detection of multiple pathogens (20).

Serological Testing: Which detects RSV-specific Immunoglobulin M (IgM) and Immunoglobulin G (IgG) antibodies, faces significant constraints. While IgM can indicate acute infection within 1–2 weeks, its utility in young infants is limited by immunological immaturity, often causing false-negative results due to delayed or low-titer responses. Like viral culture, this method is not designed for multiplex pathogen detection, restricting its role in co-infection diagnosis (19).

Bacterial Culture: The standard for confirming bacterial infections, identifies organisms and conducts antimicrobial susceptibility testing from specimens like blood or sputum. However, its sensitivity is often suboptimal, particularly if patients have received prior antibiotic therapy. The extended turnaround time of 2–3 days also limits its ability to guide timely clinical decisions (26).

### 5.2 Molecular diagnostic methods

The diagnosis of RSV and its associated co-infections has been transformed by molecular and genomic technologies, which offer superior speed, sensitivity, and comprehensiveness compared to traditional methods.

PCR: PCR-based methods have become the cornerstone of modern pathogen detection due to their high sensitivity, specificity, and relatively rapid turnaround time. Real-time PCR, in particular, allows not only for the definitive identification of RSV but also for the quantification of viral load, which can provide valuable insights into disease progression and infectiousness. In Eastern China, Cui et al. effectively employed this technology to detect RSV among a panel of respiratory viruses. The significant advancement to multiplex PCR has dramatically improved diagnostic efficiency for co-infections by enabling the simultaneous detection of multiple pathogens—such as RSV, HRV, Spn, and Hi —from a single patient sample (20). This capability is critical for accurate clinical management. Similarly, Do et al. utilized a multiplex real-time reverse transcription-polymerase chain reaction **(**RT-PCR) platform capable of identifying 13 respiratory viruses, which proved essential for diagnosing complex viral co-infections involving RSV (19). The field continues to evolve with even more rapid and portable platforms. A promising development is a novel, rapid, single-tube, dual-gene detection method for RSV and HRV. This technique integrates reverse transcription-recombinase polymerase amplification (RT-RPA)—an isothermal amplification method—with the specific target recognition of CRISPR-Cas12a and Cas13a systems. By leveraging the orthogonal trans-cleavage activities of these Cas enzymes, the assay achieves high specificity and sensitivity, presenting a potential future tool for point-of-care or near-patient testing (44).

Next-Generation Sequencing (NGS): NGS represents a paradigm shift towards unbiased, high-throughput pathogen detection. This technology can identify a vast spectrum of pathogens—viruses, bacteria, fungi, and protozoa—in a single assay without prior suspicion of a specific agent. Beyond mere detection, NGS enables genomic characterization for strain typing, epidemiological tracking, and the identification of antimicrobial resistance genes. A targeted application of this technology, targeted NGS (tNGS), uses pathogen-specific primers to enrich and sequence relevant genomic material from clinical samples, thereby increasing sensitivity for a predefined set of organisms. In Guangxi, China, Fu et al. applied tNGS to successfully achieve a rapid diagnosis of RSV and MP co-infections (29). However, the widespread clinical adoption of NGS technologies, faces considerable hurdles. These include high costs, technical complexity, lengthy data analysis requiring specialized bioinformatics expertise, and longer turnaround times compared to standard PCR. Consequently, its routine use is currently limited to reference laboratories and complex clinical cases rather than primary or acute care settings.

### 5.3 Biomarker detection

Biomarker assays provide adjunctive value in the diagnosis and severity assessment of pediatric RSV co-infections. Commonly used biomarkers include CRP, Procalcitonin (PCT), and IL-6. Do et al. (2020) observed significantly elevated PCT levels (median: 2.3 ng/mL) in children with RSV–bacterial co-infections, compared to mild or normal levels (median: 0.3 ng/mL) in those with RSV monoinfection. A PCT cutoff of >2.25 ng/mL demonstrated 92% specificity for identifying bacterial co-infection (26). Jiang et al. also reported higher CRP levels in co-infected children, which correlated with disease severity (15). Similarly, IL-6—a key inflammatory cytokine—is often elevated in co-infected patients and may assist in evaluating clinical severity (26). However, biomarkers such as CRP and PCT can also rise in non-infectious conditions; thus, their interpretation requires integration with clinical presentation and imaging findings.

In addition to CRP and PCT, Erythrocyte Sedimentation Rate (ESR) may provide supplementary diagnostic information. Available pediatric data suggest that ESR can be elevated during viral respiratory episodes, including RSV infection, but has limited pathogen specificity: in the study by Asseri et al., the mean ESR in children with RSV infection was 34.8 ± 23 mm/h, and no significant difference was observed compared with SARS-CoV-2, Influenza A, or Influenza B infection (*p* = 0.659) (36). In addition, comparative pediatric data showed that ESR was significantly lower in viral pneumonia than in MP pneumonia [14.0 [5.0–26.0] vs. 25.0 [15.5–35.0] mm/h, *p* < 0.001], suggesting that relatively lower ESR values may be more compatible with viral infection, whereas more marked ESR elevation should prompt consideration of atypical or bacterial co-infection (36, 45). Therefore, ESR should be interpreted only as an adjunctive marker in combination with clinical presentation, microbiological findings, and other inflammatory biomarkers (36, 45).

## 6 Therapeutic and preventive strategies for pediatric RSV co-infections

### 6.1 Therapeutic strategies

Antiviral Therapy: Ribavirin remains the primary antiviral agent for treating RSV infections in clinical practice. According to Cui et al., ribavirin inhibits RSV replication, reduces disease severity, and shortens illness duration. However, due to its potential teratogenic and carcinogenic risks, along with the lack of standardized dosing and treatment duration in pediatric populations, its use is generally restricted to severe cases under close medical supervision (20). Ribavirin is the only antiviral drug approved by the United States Food and Drug Administration (FDA) for the treatment of RSV infection (38), and it exhibits broad-spectrum antiviral activity against RSV. Nevertheless, ribavirin is not recommended for routine use in all pediatric RSV infections. Based on available evidence, its potential benefit appears to be limited primarily to selected severe or high-risk patients, particularly immunocompromised individuals such as hematopoietic stem cell transplant recipients, solid organ transplant recipients, and patients with hematologic malignancies (20, 38, 40). Conversely, evidence does not support routine administration in otherwise healthy children with typical RSV bronchiolitis (39, 46), and its indication in pediatric practice remains controversial (39, 40).

Antibiotic Therapy: Prompt antibiotic treatment is essential for children with RSV–bacterial co-infections. In a 2020 study by Do et al., antibiotics such as amoxicillin-clavulanate and ceftriaxone were administered based on bacterial culture and sensitivity results, effectively controlling secondary bacterial infections (26). Similarly, Lin et al. (2022) recommended penicillin G or amoxicillin for penicillin-sensitive Streptococcus pneumoniae co-infections, and third-generation cephalosporins such as ceftriaxone or cefotaxime for penicillin-resistant strains (27). It is critical that antibiotic use follows medical guidance to minimize the risk of antimicrobial resistance.

Symptomatic and Supportive Care: Symptomatic and supportive management constitutes a fundamental component of treatment for RSV co-infections. Modalities include oxygen therapy, respiratory support, and fluid management. In a study from Qatar, Janahi et al. reported that oxygen delivery via nasal cannula or face mask effectively alleviated hypoxemia in co-infected children with respiratory distress (22). For those progressing to respiratory failure, non-invasive or invasive mechanical ventilation may be required to maintain adequate gas exchange (26). Additionally, maintaining fluid and electrolyte balance is crucial to prevent dehydration and metabolic disturbances. Symptomatic relief for fever, cough, and wheezing may be achieved using antipyretics, antitussives, and bronchodilators as appropriate (20).

### 6.2 Preventive strategies

Nirsevimab, a long-acting monoclonal antibody, confers immediate passive immunity via a single intramuscular injection administered prior to the RSV season onset, delivering protection for at least 150 days (47). Approved and utilized in multiple countries to prevent RSV infections and their associated severe outcomes, recent real-world data demonstrate its efficacy in safeguarding infants against RSV-related hospitalizations. Specifically, in infants aged 0–12 months, nirsevimab was linked to significantly lower odds of RSV-associated hospitalization [OR 0.17; 95% confidence interval (CI) 0.12–0.23; I^2^ = 85.8%], ICU admission (OR 0.19; 95% CI 0.12–0.29; I^2^ = 55.6%), and LRTI incidence (OR 0.25; 95% CI 0.19–0.33; I^2^ = 35.1%) (48, 49). A Japanese study indicated that the preventive effect of nirsevimab against RSV infection persisted for up to 12 months post-administration (50). A pairwise meta-analysis focusing on infants aged ≤12 months reported 76% efficacy of nirsevimab in reducing RSV-related hospitalizations (OR 0.24, 95% CI 0.13–0.47) (46). Another meta-analysis encompassing four prelicensure and six postlicensure studies estimated its effectiveness against RSV-related hospitalization at 88.4% (43). Canadian research showed that universal nirsevimab immunization could substantially alleviate the health and economic burden of RSV among Canadian infants (51). Meanwhile, an Italian study demonstrated that high nirsevimab coverage significantly reduced RSV-related hospitalizations among infants in Tuscany during the 2024–2025 season (52).

However, the global rollout of nirsevimab is marked by significant disparities. In developed nations such as those in Europe and the United States, it has been integrated into NIPs with large-scale administration, whereas the vast majority of infants in developing countries—particularly in Africa—remain entirely without access. This “immunization gap” is primarily driven by substantial cost barriers [over $400 per dose in high-income countries, far exceeding the affordability of low- and middle-income countries (LMICs)] and health system mismatches (e.g., the lack of robust primary healthcare networks necessary for seasonal immunization campaigns). Furthermore, global implementation faces shared challenges, including potential viral selective pressure from widespread monoclonal antibody use, insufficient long-term efficacy data in special populations, and issues of service accessibility and equity even in resource-rich settings. To address these inequities, the international community is advocating for tiered pricing, supporting the development of more cost-effective alternatives, and assisting LMICs in strengthening their domestic health systems and surveillance capacities.

Vaccination serves as a fundamental strategy for preventing RSV infection and its associated co-infections. The pneumococcal conjugate vaccine (PCV), as outlined by Besteman et al., effectively reduces the incidence of Spn infection, thereby lowering the risk of RSV–pneumococcal co-infection (12). Supporting this, Jiang et al. confirmed that PCV-vaccinated children experience significantly lower rates of such co-infection compared to unvaccinated individuals (15). Similarly, influenza vaccination contributes to mitigating the risk of co-infection between RSV and influenza virus (12). Recent progress in vaccine development has also yielded several promising RSV-specific candidates currently in clinical trials, indicating potential for broader prevention in the near future.

Complementing immunization, personal protective measures are vital for reducing transmission risk. Cui et al. recommend that parents educate children on essential hygiene practices—including frequent handwashing and avoiding touching the face—to limit the spread of pathogens (20). During periods of high RSV activity, it is advisable for children to avoid crowded and poorly ventilated public spaces, such as shopping malls and indoor playgrounds, to minimise exposure (22). Furthermore, improving indoor ventilation and maintaining clean home environments can help reduce the concentration of respiratory pathogens in household settings.

In healthcare environments, stringent infection control is crucial given the high risk of RSV transmission. Janahi et al. emphasise the importance of establishing robust infection prevention protocols and training healthcare workers in RSV-specific containment strategies (22). Medical staff should adhere to strict hand hygiene and use appropriate personal protective equipment, including masks and gloves, when caring for infected children. Regular disinfection of patient rooms, clinical surfaces, and medical equipment is also essential to minimise environmental contamination (20). Where feasible, isolating patients who test positive for RSV can further help limit nosocomial spread.

## 7 Discussion

This review synthesizes the evolving landscape of pediatric RSV co-infections in the wake of the COVID-19 pandemic. Our analysis confirms that RSV co-infection is a frequent and consequential clinical reality, with an estimated pooled prevalence of approximately 30%. HRV emerges consistently as the predominant viral partner, while Spn and Hi are the leading bacterial co-pathogens. Critically, the presence of these co-infections is not merely coincidental but is significantly associated with a more severe disease phenotype. This is evidenced by exacerbated clinical symptoms, prolonged hospital stays, increased demands for intensive care and respiratory support, and greater therapeutic complexity, findings that align with and extend previous reports from diverse geographical settings.

The post-pandemic period has introduced unprecedented volatility into RSV epidemiology, characterized by off-season surges, altered peak intensities, and a notable shift in the age distribution of severe cases toward older infants and young children. This review documents that the relaxation of NPIs has been a key driver of this disruption, facilitating a rebound in RSV circulation and, consequently, co-infection rates. The phenomenon of “immunity debt” resulting from reduced pathogen exposure during strict NPI periods, likely contributed to the size of the susceptible pediatric population, fueling these atypical outbreaks. These observations underscore that RSV epidemiology is dynamically influenced by public health measures and population immunity, necessitating flexible and responsive surveillance systems.

Advancements in molecular diagnostics, particularly multiplex PCR, have been instrumental in delineating the true scope and microbial composition of co-infections, moving beyond the limitations of traditional culture methods. The concurrent development of effective immunoprophylaxis, most notably the long-acting monoclonal antibody nirsevimab, represents a transformative step in prevention. However, the emerging “immunization gap” and the high cost of novel biologics threaten to exacerbate global health inequities, potentially leaving the most vulnerable children in resource-limited settings without protection. Therefore, while our diagnostic and preventive toolkit is expanding, translating these advancements into equitable health gains remains a paramount challenge.

This review has several limitations that should be considered when interpreting its findings. First, as a narrative synthesis, it may be subject to selection bias, as included studies were not identified through a formal, exhaustive systematic review process. Second, the evidence base is heavily weighted toward hospital-based studies, which captures the more severe end of the disease spectrum and may not reflect the full picture of co-infection epidemiology in the community. Third, the heterogeneity in study methodologies, including differences in diagnostic panels (e.g., the number of pathogens tested for), patient inclusion criteria, and definitions of disease severity, complicates direct comparisons and pooled quantitative analysis across reports. Finally, the post-pandemic era is still evolving. Data on the long-term stability of the observed epidemiological shifts, the durability of immune protection from new prophylactics, and the potential for viral or bacterial strain evolution under changed epidemiological pressures are still maturing.

Future research should focus on several priorities. First, mechanistic studies are needed to clarify the pathogenesis of RSV co-infections. Second, more sensitive, specific, and accessible diagnostic approaches should be developed, alongside broader implementation of multiplex PCR panels and other rapid molecular assays to enable simultaneous detection of RSV and co-pathogens, earlier etiologic identification, and expansion of near-patient or point-of-care testing (19, 38, 44). Third, prompt microbiological diagnosis should be better integrated into clinical practice to facilitate earlier pathogen-directed treatment, including timely targeted antibiotic therapy or other appropriate interventions when bacterial or atypical co-infection is suspected (17, 26, 27, 29, 38). In parallel, continued development of safer and more effective therapies, next-generation vaccines, and evidence-based prevention and control strategies remains essential. Strengthened international collaboration and data sharing will also be critical to addressing pediatric RSV co-infections as an important global public health challenge and to improving long-term outcomes in affected children worldwide.
