# Supplementary material for: Epidemiology and clinical impact of pediatric RSV co-infections after the COVID-19 pandemic: a narrative review
Source: Front Pediatr. 2026 Apr 24;14:1787312. doi: 10.3389/fped.2026.1787312 (PMC13153121; doi:10.3389/fped.2026.1787312)
Supplement: Supplementary file 1 [file Supplementaryfile1.docx]

Supplementary Material

**Epidemiology and Clinical Impact of Pediatric RSV Co-infections after the COVID-19** **Pandemic: a narrative review**

Yingying Hu^1^**^,^**^*^, Jun Li^1^**^,^**^*^,Yixiang Zheng^1^**^,^**, Youde Cheng^1^**^,^**,Wei Li^2^**^,^**, Xu Wang^2^**^,^** ^†^, Yanqun Sun^2, †^

^1^Department of Pediatrics, Affiliated Nanjing Jiangbei Hospital of Xinglin College, Nantong University, Nanjing, China.

^2^Clinical Medical Research Center, Children’s Hospital of Nanjing Medical University, Nanjing, China.

***** **These authors contributed equally to this work.**

**† Correspondence to:**

Xu Wang: sepnine@njmu.edu.cn

Yanqun Sun: [yanq_sun@163.com](mailto:yanq_sun@163.com)

**Table A1** **Summary of RSV Infection-related Literature Information Before and After the COVID-19 Pandemic**

| **Publication Year** | **Country** | **Inclusion Period** | **Inclusion Age** | **Number of Included Cases** **(cases)** | **RSV Positive Cases** **(cases)** | **Detection Method** | **RSV Co-infection Cases** **(cases)** | **Viruses Frequently Co-infected with RSV** | **Bacteria/Other Pathogens Frequently Co-infected with RSV** | **The outcome of RSV co-infection** |
| --- | --- | --- | --- | --- | --- | --- | --- | --- | --- | --- |
| 2025^1^ | Denmark | 2021.8-2021.12 | ≤5y | 148 | 66 | PCR | 34 | HEV | \ | Elevated CRP and antibiotic usage ratio; prolonged treatment duration. |
| 2025^2^ | Portugal | 2021.4-2023.1 | ≤2y | 678* | 678 | PCR | 138 | \ | Hi, Spn | Higher ICU admission rate, longer hospital stay, more respiratory support |
| 2025^3^ | Zhejiang, China | 2018.1-2023.8 | ≤5y | 7857* | 7857 | VA,AD | 1601 | HRV(437)  CMV(381)HAdV(224) | MP(947);  Spn(405); Hi(140) | Higher hospitalization costs |
| 2025^4^ | Wenzhou, China | 2021.12-2023.4 | ≤14y | 1063* | 1063 | RT-PCR | 502 | HRV(211) | Spn(187) | Longer hospital stay, higher rate of abnormal chest X-rays |
| 2025^5^ | Zhejiang, China | 2022 | <18y | 12993 | 917 | RT-PCR | 153 | HRV (5) | \ | \ |
| 2025^5^ | Zhejiang, China | 2023 | <18y | 39019 | 3612 | RT-PCR | 923 | \ | MP (232) | \ |
| 2025^6^ | Henan,China | 2015.1-2023.5 | <15y | 183771 | 22044 | RT-PCR | 3976 | PIV (641)  PIV+Flu B (46) | \ | \ |
| 2024^7^ | Hebei, China | 2018.6-2019.1 | <2y | 82* | 82 | real-time RT-qPCR | 20 | 7 | 11 | Increased severe disease rate |
| 2024^8^ | Taiwan, China | 2007.1-2018,12 | <18y | 1985* | 1985 | VIC, PCR, RAT | 802 | \ | \ | Increased severe disease rate |
| 2024^9^ | \ | ~2024.3 | All age | \ | \ |  | \ | \ | \ | RSV and Spn have a synergistic pathogenic interaction |
| 2023^10^ | Belgium | 2018.10-2020.2 | 28d-2y | 120 | 110 | RT-PCR | 47 | HAdV(10), HRV(9) | \ | \ |
| 2023^11^ | Madrid, Spain | 2020.3-2023.1 | <2y | 597* | 597 | RT-PCR | 13 | COVID-19 | \ | \ |
| 2022^12^ | Taiwan, China | 2010.1-2019.12 | <18y | 620* | 620 | RT-PCR | 201 | \ | Spn(26), SA(26), Hi(16) | Increased ventilator use and ICU admission; prolonged hospitalization |
| 2021^13^ | Chile | \ | \ | \ | \ | \ | \ | \ | \ | Longer duration of mechanical ventilation |
| 2020^14^ | \ | ~2019.12 | <5y | \ | \ | \ | \ | \ | \ | Higher ICU admission rate, longer hospital stay |
| 2020^15^ | Vietnam | 2015.1-2017.3 | <5y | 70* | 70 | \ | 11 | \ | Hi, KP, PA | More pronounced clinical symptoms, abnormal test indicators, and a higher mortality rate |
| 2018^16^ | Rome, Italy | 2004-2016 | <1y | 486 | 365 | RT-PCR | 45 | HRV(20), HBoV(18) | \ | Higher prevalence of asthma family history |
| 2017^17^ | Suzhou,China | 2015.1-2015.12 | 1m-14y | 846 | 194 | DFA, PCR | 293 | HRV(142), HBoV (67) | \ | Higher PICU admission rate |
| 2017^18^ | Madrid, Spain | 2008.9-2011.12 | 6-8y | 244 | 100 | RT-PCR | 52 | HRV(15), HBoV(12) | \ | The strongest independent risk factor for asthma in children aged 6-8 years |
| 2016^19^ | Finland | 2008-2010 | ≤2y | 4810 | 279 | RT-PCR | 18^#^ | HRV(18) | \ | A longer hospital stay |
| 2016^20^ | Vietnam | 2009.5-2010.12 | 1m-2y | 632 | 302 | RT-PCR |  | HRV, HEV 和 HAdV | \ | \ |
| 2016^21^ | Eastern China | 2009.1-2013.12 | All age | 1046* | 1046 | RT-PCR | 390 | HInV (130) | \ | Increased incidence of sore throat and dyspnea |
| 2015^22^ | Spain | 2005.9-2013.8 | <14y | 2993 | 599 | RT-PCR | 326 | \ | \ | Fever, hypoxia, and an increase in ICU admission rate |
| 2024^23^ | Kunming,China | 2019.1-2019.12 | ≤14y | 331* | 331 | \ | 107 | HRV,MPV,MP | \ | Higher rate of severe illness |
| 2024^23^ | Kunming,China | 2023.1-2023.12 | ≤14y | 628* | 628 | \ | 252 | MP,FluA,HRV | \ | Higher rate of severe illness |
| 2022^24^ | Suzhou,China | 2017.1-2019.12 | ≤2y | 280* | 280 | RT-PCR | 113 | \ | Spn, Hi, SA, Mrx | Higher rate of severe illness |
| 2025^25^ | Suzhou,China | 2021.7-2023.7 | <18y | 1178 | 299 | PCR | 183 | 37 | 70, MP(27) | More severe clinical symptoms |
| 2025^26^ | Argentina | 2019-2023 | ≤2y | 401 | 172 | RT-PCR | 159 | \ | Hi(117),Spn(111),Mrx(90) | Higher rate of severe illness |
| 2025^27^ | Hebei, China | 2019.1-2023.12 | 1m-18y | 43978 | 11424 | RT-PCR | 4054 | HRV(1714) | \  Spn(342)；MP(301) | \ |
| 2025^28^ | Hubei,China | 2017.9-2024.8 | <14y | 105264 | 12407 | \ | \ | \ | \ | \ |
| 2025^29^ | Nanjing,China | 2024.9-2024.12 | 3m-15y | 543 | 39 | RT-RPA-Cas13a/Cas12a | 6^#^ | HRV(6) | \ | \ |
| 2025^30^ | Qingdao,China | 2023.3-2023.5 | <1y | 95 | 34 | BTC, ITAF, fluorescent PCR, tNGS | 10 | \ | \ | \ |
| 2025^31^ | Wuhan,China | 2019.3-2023.1 | ≤6y | 12743 | 715 | PCR | 110 | HRV(61), MPV(18) | \ | \ |
| 2025^32^ | Malaysia | 2022.4-2023.3 | All age | 441 | 82 | MLA,PSA | \ | HRV(26) | \ | \ |
| 2025^33^ | Taiwan, China | 2021.1-2022.12 | <18y | 1983 | 279 | PCR | 26 | COVID-19 | \ | \ |
| 2025^34^ | Hefei,China | 2021.2-2021.4 | ≤10y | 438 | 50 | PCR | 15 | HRV(9), HAdV(5) | \ | Higher rate of severe illness |
| 2025^35^ | Laos | 2021.3-2023.7 | All age | 4203 | 402 | RT-PCR | 33 | FluB(17), COVID-19(8), FluA(5) | \ | \ |
| 2025^36^ | Iran | 2021.10-2022.3 | <5y | 264 | 48 | PCR | 30 | HMPV | \ | \ |
| 2025^37^ | Shandong,China | 2023.11-2024.1 | All age | 2490 | 251 | PCR-Fluorescent Probe Method | 85 | HAdV(27), HRV(18), MP(16) | \ | \ |
| 2025^38^ | Chongqing,China | 2023.4-2025.3 | <18y | 7627 | 1263 | qPCR | \ | HRV(14.19%), HAdV(4.79%), FluA(4.38%) | \ | \ |
| 2025^39^ | Guangzhou,China | 2018.1-2023.12 | <18y | 317828 |  | qPCR | 749 | HAdV(192)HBoV(133)MP(133) | \ | \ |
| 2025^40^ | Shanghai,China | 2017.1-2022.12 | All age | 24933 | 60 | MIFA | 29 | MP(17) |  | \ |
| 2025^41^ | Shanghai,China | 2020.3-2024.11 | All age | 748 | \ | qPCR | 3 | HEV, FluA, HAdV | \ | \ |
| 2025^42^ | India | 2022.4-2023.3 | ≤2y | 390 | 49 | rRT-PCR | 10 | FluA(2) | Hi(4), Spn(2) | \ |
| 2025^43^ | Tanzania | 2022.3-2022.5 | <14y | 475 | 35 | rRT-PCR | 15 | HBoV(8), HEV(4) | \ | \ |
| 2025^44^ | Yongzhou,China | 2019.1-2024.6 | All age | 4626 | 279 | RT-PCR | \ | HAdV(143), COVID-19(7) | \ | \ |
| 2025^45^ | Taiwan, China | 2018.9-2023.8 | All age | 14957 | 300 | VIC, AD | 40 | HInV(4), HEV(2) | Mrx(10), Hi(9), Spn(5) | \ |
| 2025^46^ | Iran | 2021.12-2022.3 | <18y | 92 | 2 | PCR/RT-PCR | \ | HAdV(2), CMV(1) | \ | \ |
| 2025^47^ | Suzhou,China | 2013.1-2023.12 | 0-28d | 7420 | 875 | PCR, ELISA, RT-qPCR | 229 | PIV(7), | SA(89), E. coli(37), KP(24), MP(22), CP(12) | Increased LRTI mortality |
| 2025^48^ | Mexico | 2021.1-2022.12 | <19y | 3619 | 475 | qPCR | 151 | HRV | \ | \ |
| 2025^49^ | Lebanon | 2018.9-2022.12 | <18y | 1069 | 147 | RIDT, RAT | 1 | FluA(1) | \ | \ |
| 2025^50^ | Mexico | 2010.3-2013.8 | <5y | 1404 | 531 | multiplex RT-qPCR | 149 | \ | \ | \ |
| 2025^50^ | Mexico | 2021.7-2023.3 | <5y | 579 | 248 | multiplex RT-qPCR | 207 | HRV(133), HBoV(17), MPV(6) | \ | \ |
| 2025^51^ | Rome, Italy | 2022.1-2024.4 | <18y | 17259 | 952 | RT-PCR | 351 | HRV(220), HBoV(70), HEV(55) | \ | \ |
| 2025^52^ | Praia, Cape Verde, Spain | 2022.1, 5, 11月 | <5y | 96 | 21 | multiplex PCR | 6 | HRV(4) | \ | Rise in post-pandemic co-infections |
| 2025^53^ | Suzhou,China | 2016.11-2017.3 | <2y | 89 | 34 | DFA, PCR, RT-PCR | 2 | HRV(2) | \ | \ |
| 2025^54^ | Gansu,China | 2023.4-2024.3 | <13y | 9670 | 365 | real-time PCR | \ | HRV(28), HAdV(10), | \ | \ |
| 2025^55^ | Turkey | 2017-2019, 2022-2024 | <5y | 541 | 116 | multiplex PCR | \ | \ | \ | Higher demands for intensive care |
| 2025^56^ | Tokyo, Japan | 2020.11-2023.3 | All age | 57746 | 477 | multiplex PCR | 180 | \ | \ | Higher incidence of severe BA |
| 2025^57^ | United Arab Emirates | 2018.1-2022.12 | ≤5y | 39760 | 2559 | RAT, multiplex PCR | 182 | HAdV(106), HEV(38), PIV3(17) | \ | A longer hospital stay |
| 2025^58^ | New South Wales, Australia | 2018.1-2024.12 | All age groups | 370126 | 13293 | RT-qPCR | 2713 | HRV(1003), HBoV(461), HAdV(298) | \ | \ |
| 2025^59^ | Fujian,China | 2023.1-2023.12 | <18y | 22769 | 2531 | multiplex PCR | 262 | \ | \ | \ |
| 2025^60^ | Shenzhen,China | 2020.1-2023.12 | <18y | 53033 | 6830 | multiplex PCR | 1144 | \  HRV(600), PIV(118), MP(113) | \ | \ |
| 2025^61^ | America | 2016.12-2020.3 | <2y | 18008 | 5099 | RT-PCR | 1172 | HRV(496), HAdV (204), HCoV (151) | \ | \ |

*: The included cases were all patients who were RSV positive. #: This study only examined RSV and HRV. LRTI: Lower Respiratory Tract Infections. BA: Bronchial Asthma. HBoV:Human Bocavirus. PIV3: Parainfluenza Virus 3. HInV: Human influenza virus. HEV:Human Enterovirus. MP:Mycoplasma pneumoniae. Flu A:Influenza A virus. COVID-19:Coronavirus disease 2019. Flu B:Influenza B virus. MPV:Human Metapneumovirus. HCoV:Human coronavirus. HAdV:Human adenovirus. CMV:cytomegalovirus. HRV:Human Rhinovirus. CP:Chlamydia pneumoniae. KP:Klebsiella pneumoniae. E. coli:Escherichia coli. Mrx:Moraxella catarrhalis. SA:Staphylococcus aureus. Hi :Haemophilus influenzae. Spn:Streptococcus pneumoniae. MP:Mycoplasma pneumoniae. BALF:bronchoalveolar lavage fluid. PCR:polymerase chain reaction. RT-PCR:reverse transcription polymerase chain reaction. fluorescent PCR:fluorescent polymerase chain reaction. AD:antigen detection. VA:vianucleic acid. rRT-PCR:real-time reverse transcription polymerase chain reaction. VIC:viral culture. RAT:rapid antigen test. DFA:direct immunofluorescence assay. RT-RPA:reverse transcription-recombinase polymerase amplification. BTC:bacterial culture. ITAF:isothermal amplification. tNGS:targeted next generation sequencing (tNGS). MS:molecular assays. PSA:pan-species assays. qPCR:quantitative polymerase chain reaction. MIFA:multiple indirect immunofluorescence assay. RIDT:Rapid Influenza Diagnostic Test. multiplex RT-PCR:multiplex reverse transcription polymerase chain reaction. multiplex PCR:multiplex polymerase chain reaction. real-time PCR:real-time polymerase chain reaction

**References**

1. Vahlkvist S, Mohammad A, Kofoed P. The Impact of Viral Co‐Infection in Children Treated With Respiratory Support Due to Lower Respiratory Tract Infections. An Observational Study. *Pediatric Pulmonology*. 2025;60(1):e27467. doi:10.1002/ppul.27467.
2. Torres AR, Gaio V, Melo A, et al. RSV-Bacterial Co-Infection Is Associated With Increased Illness Severity in Hospitalized Children - Results From a Prospective Sentinel Surveillance Study. *J Med Virol*. 2025;97(2):e70209. doi:10.1002/jmv.70209.
3. Sun W, Zhu A, Zhu Z, et al. Multicenter Study on the Prevalence of Human Respiratory Syncytial Virus Coinfection and Disease Burden Among Hospitalized Children Aged 5 Years and Younger - 5 Prefecture-level Cities, Zhejiang Province, China, 2018-2023. *China CDC Weekly*. 2025;7(4):137-143. doi:10.46234/ccdcw2025.021.
4. Qu X, Ye X, Yu J, et al. Epidemiological and clinical characteristics of bacterial co-detection in respiratory syncytial virus-positive children in Wenzhou, China, 2021 to 2023. *BMC Infect Dis*. 2025;25(1):697. Published 2025 May 14. doi:10.1186/s12879-025-11086-z.
5. Lai QR, Chu XL, Chen YY, Li W, Guo YJ, Shang SQ. Epidemiological and Clinical Characteristics of Respiratory Syncytial Virus Infection in Children in Hangzhou (2022–2023). *Pathogens*. 2025;14(6):603. doi:10.3390/pathogens14060603.
6. Dai B, Chen C, Shen Y, et al. Hospitalizations trends and co-infection patterns for respiratory virus infections in children in central China 2015 to 2023: a longitudinal surveillance study. *BMC Infect Dis*. 2025;25(1):1058. doi:10.1186/s12879-025-11352-0.
7. Yang S, Lu S, Wang Y, et al. Respiratory syncytial virus subtypes in children with bronchiolitis: does it correlate with clinical severity?. *BMC Infect Dis*. 2024;24(1):263. doi:10.1186/s12879-024-09129-y.
8. Ma HY, Lin IF, Liu YC, et al. Risk Factors for Severe Respiratory Syncytial Virus Infection in Hospitalized Children. *Pediatric Infectious Disease Journal*. 2024;43(6):487-492. doi:10.1097/INF.0000000000004270.
9. Besteman SB, Bogaert D, Bont L, et al. Interactions between respiratory syncytial virus and Streptococcus pneumoniae in the pathogenesis of childhood respiratory infections: a systematic review. *The Lancet Respiratory Medicine*. 2024;12(11):915-932. doi:10.1016/S2213-2600(24)00148-6.
10. Stobbelaar K, Mangodt TC, Van der Gucht W, et al. Risk Factors Associated with Severe RSV Infection in Infants: What Is the Role of Viral Co-Infections?. *Microbiol Spectr*. 2023;11(3):e0436822. doi:10.1128/spectrum.04368-22.
11. Rodriguez-Fernandez R, González-Martínez F, Perez-Moreno J, et al. Clinical Relevance of RSV and SARS-CoV-2 Coinfections in Infants and Young Children. *Pediatric Infectious Disease Journal*. 2023;42(12):e473-e475. doi:10.1097/INF.0000000000004080.
12. Lin HC, Liu YC, Hsing TY, et al; RSV pneumonia with or without bacterial co-infection among healthy children. *Journal of the Formosan Medical Association*. 2022;121(3):687-693. doi:10.1016/j.jfma.2021.08.012.
13. Pacheco GA, Gálvez NMS, Soto JA, Andrade CA, Kalergis AM. Bacterial and Viral Coinfections with the Human Respiratory Syncytial Virus. *Microorganisms*. 2021;9(6):1293. doi:10.3390/microorganisms9061293.
14. Li Y, Pillai P, Miyake F, Nair H; The role of viral co-infections in the severity of acute respiratory infections among children infected with respiratory syncytial virus (RSV): A systematic review and meta-analysis. *Journal of Global Health*. 2020;10(1):010426. doi:10.7189/jogh.10.010426.
15. Do Q, Dao TM, Nguyen TNT, Tran QA, Nguyen HT, Ngo TT; Procalcitonin Identifies Bacterial Coinfections in Vietnamese Children with Severe Respiratory Syncytial Virus Pneumonia. *BioMed Research International*. 2020;2020(1):7915158. doi:10.1155/2020/7915158.
16. Petrarca L, Nenna R, Frassanito A, et al. Acute bronchiolitis: Influence of viral co‐infection in infants hospitalized over 12 consecutive epidemic seasons. *Journal of Medical Virology*. 2018;90(4):631-638. doi:10.1002/jmv.24994.
17. Jiang W, Wu M, Zhou J, et al. Etiologic spectrum and occurrence of coinfections in children hospitalized with community-acquired pneumonia.  *BMC Infect Dis*. 2017;17(1):787. doi:10.1186/s12879-017-2891-x.
18. Garcia-Garcia ML, Calvo C, Ruiz S, et al. Role of viral coinfections in asthma development. *PLoS One*. 2017;12(12):e0189083. doi:10.1371/journal.pone.0189083.
19. Karppinen S, Toivonen L, Schuez-Havupalo L, Waris M, Peltola V. Interference between respiratory syncytial virus and rhinovirus in respiratory tract infections in children. *Clinical Microbiology and Infection*. 2016;22(2):208.e1-208.e6. doi:10.1016/j.cmi.2015.10.002.
20. Do LA, Bryant JE, Tran AT, et al. Respiratory Syncytial Virus and Other Viral Infections among Children under Two Years Old in Southern Vietnam 2009-2010: Clinical Characteristics and Disease Severity. *PLoS One*. 2016;11(8):e0160606. doi:10.1371/journal.pone.0160606.
21. Cui D, Feng L, Chen Y, et al. Clinical and Epidemiologic Characteristics of Hospitalized Patients with Laboratory-Confirmed Respiratory Syncytial Virus Infection in Eastern China between 2009 and 2013: A Retrospective Study. *PLoS One*. 2016;11(11):e0165437. doi:10.1371/journal.pone.0165437.
22. Calvo C, García-García ML, Pozo F, et al. Respiratory Syncytial Virus Coinfections With Rhinovirus and Human Bocavirus in Hospitalized Children. *Medicine*. 2015;94(42):e1788. doi:10.1097/MD.0000000000001788.
23. Liu HF, Feng QL, Huang RW, et al. Clinical characteristics and risk prediction of severe disease in hospitalized children with respiratory syncytial virus infection in Kunming during the post-COVID-19 pandemic period. *Chin J Pediatr*. 2024;62(4):323-330. doi: 10.3760/cma.j.cn112140-20240219-00109.
24. Zhang J, Sun HM, Li SX, et al. A study on nasopharyngeal bacterial categories and clinical characteristics in children with bronchiolitis infected by respiratory syncytial virus. *Chin J Appl Clin Pediatr*. 2022;37(10):738-742. doi:10.3760/cma.j.cn101070-20210315-00306.
25. Zhou LT, Chen SN, Xu XY, et al. Clinical characteristics and peripheral blood cell analysis of mixed infection in hospitalized children with respiratory syncytial virus lower respiratory tract infection. *Chin J Hemorheol*. 2025;35(1):138-143. doi:10.3969/j.issn.1009-881X.2025.01.029.
26. López EL, Ferolla FM, Denardi AF, et al. Bacterial colonization and life-threatening respiratory syncytial virus infection in children. *Journal of Clinical Virology*. 2026;182:105891. doi:10.1016/j.jcv.2025.105891.
27. Wang X, Lu SK, Liu JH, et al. Epidemiological characteristics of respiratory syncytial virus infection in children in Hebei region. *Chin J Contemp Pediatr*. 2025;27(10):1199-1204. doi:10.7499/j.issn.1008-8830.2502033.
28. Zhou Y, Xia JB, Luo M, et al. Shifts in epidemic patterns and age dynamics of RSV, IFV, and SARS-CoV-2 in Chinese children following COVID-19 non-pharmaceutical intervention relaxation, 2017 - 2024. *Eur J Clin Microbiol Infect Dis*. Published online October 13, 2025. doi:10.1007/s10096-025-05249-8.
29. Zhao X yi, Gao C, Zhao W wu, et al. Development of a single-tube, dual-target CRISPR Cas12a/Cas13a system for rapid screening of coinfection with respiratory syncytial virus and rhinovirus. *Virol J*. 2025;22(1):311. doi:10.1186/s12985-025-02938-w.
30. Xue A, Wei Q, Liang J, et al. Detection of respiratory pathogens in infants using targeted next-generation sequencing versus conventional methods in Qingdao. *BMC Infect Dis*. 2025;25(1):1464. doi:10.1186/s12879-025-11839-w.
31. Wu Z, Ye C, Wang G, et al. Seasonal patterns and prevalence of respiratory pathogens in children with acute respiratory infections in Wuhan, China. *J Infect Dev Ctries*. 2025;19(06):825-832. doi:10.3855/jidc.19373.
32. Toh TH, Lee JSY, Yong SM, et al. Co-infections with Multiple Viruses: A Frequent cause of Community-Acquired Pneumonia in Sarawak Malaysia. *IJID Regions*. 2025;17:100748. doi:10.1016/j.ijregi.2025.100748.
33. Tai IH, Hsiao CT, Chu CH, Tsai WJ, Chen YJ, Wu HP. Co-detection of various viruses in SARS-CoV-2 in children with respiratory infections. *Sci Rep*. 2025;15(1):8312. doi:10.1038/s41598-025-92878-w.
34. Shi YR, Yuan K, Yue L, et al. Clinical characteristics of single human rhinovirus infection and co-infection in the respiratory tract of children. *Transl Pediatr*. 2025;14(3):373-381. doi:10.21037/tp-24-79.
35. Phommasone K, Chommanam D, Christy NC, et al. Influenza and respiratory syncytial virus dynamics in Lao PDR during the COVID-19 pandemic: a hospital-based surveillance studyCOVID-19. *BMJ Open*. 2025;15(9):e098006. doi:10.1136/bmjopen-2024-098006.
36. Nateghi A, Hamrahjoo M, Yasaghi M, et al. Epidemiology and Clinical Features of Respiratory Viruses in Hospitalized Iranian Children During the COVID‐19 Pandemic. *Immun Inflamm Dis*. 2025;13(9):e70275. doi:10.1002/iid3.70275.
37. Ma H, Hou J, Wang J, Yuan Z, Man S. Analysis of common respiratory pathogens and epidemiological trends during peak influenza seasons in Tengzhou. *BMC Infect Dis*. 2025;25(1):845. doi:10.1186/s12879-025-11146-4.
38. Luo C, Wang X, Zhou N. Epidemiological characteristics of pediatric respiratory pathogens and their association with climate in Wuxi, Chongqing, China. *New Microbes and New Infections*. 2025;68:101656. doi:10.1016/j.nmni.2025.101656.
39. Liang DF, Guo WL, Zhu DP, et al. Changes in the epidemic patterns of respiratory pathogens of children in guangzhou, China during the COVID-19 pandemic. *BMC Infect Dis*. 2025;25(1):833. doi:10.1186/s12879-025-11215-8.
40. Li X, Wang J, Dai J, et al. Comparison of respiratory pathogen infections in hospitalized patients before and during the COVID-19 pandemic in Shanghai, China. Prokesch BC, ed. *Microbiol Spectr*. 2025;13(11):e00435-25. doi:10.1128/spectrum.00435-25.
41. Koçer İ, Demirbakan H, Aktaş A. Temporal dynamics and forecasting of respiratory viral infections during and after the SARS-CoV-2 pandemic (2020–2027): a multiplex PCR and ARIMA-based study. *Front Microbiol*. 2025;16:1674529. doi:10.3389/fmicb.2025.1674529.
42. Khan T, Halder S, Das RS, et al. Molecular epidemiology of influenza, respiratory syncytial virus, SARS-CoV-2, other respiratory viruses and bacteria among children 0–2-year-olds in West Bengal: a one-year influenza-like illness surveillance study (2022–2023). *Front Epidemiol*. 2025;5:1578951. doi:10.3389/fepid.2025.1578951.
43. Kelly ME, Msafiri F, Averhoff F, et al. Non-Influenza and Non-SARS-CoV-2 Viruses Among Patients with Severe Acute Respiratory Infections in Tanzania: A Post-COVID-19 Pandemic Snapshot. *Viruses*. 2025;17(8):1042. doi:10.3390/v17081042.
44. Jiang Y, Lu J, Tan Z, et al. Epidemiological characteristics of acute viral and mycoplasma respiratory infections in Yongzhou, China: a retrospective descriptive study. *Front Public Health*. 2025;13:1614985. doi:10.3389/fpubh.2025.1614985.
45. Hu Y, Ho S, Cheng A, Huang Y, Fang C, Chang L. Comparison of Clinical Features and Severity of Subgroup A and B Respiratory Syncytial Virus Infection. *Journal of Medical Virology*. 2025;97(6):e70453. doi:10.1002/jmv.70453.
46. Hosseinpour Sadeghi R, Pourakbari B, Mahmoudi S, et al. Evaluation of common respiratory viruses other than SARS-CoV-2 in hospitalized children during the COVID-19 pandemic. *BMC Infect Dis*. 2025;25(1):910. doi:10.1186/s12879-025-11293-8.
47. Gu F, Yu T, Li Q, Xu J, Lu L. Epidemiological and clinical characteristics of respiratory syncytial virus infection in hospitalized neonates in Suzhou. *Transl Pediatr*. 2025;14(10):2657-2666. doi:10.21037/tp-2025-414.
48. Flores-Alanis A, Rodríguez-Martínez G, Saldaña-Ahuactzi Z, et al. Demographic and clinical factors are relevant in respiratory infections among paediatric patients: a cross-sectional study in Mexico. *Eur J Clin Microbiol Infect Dis*. 2025;44(8):1995-2005. doi:10.1007/s10096-025-05168-8.
49. Eid R, Sayad A, Samaan W, Salameh P, Farah RA. Prevalence of influenza A and B and respiratory syncytial virus infections before and during COVID-19 pandemic in the pediatric population in Lebanon: A retrospective study. Anjorin AA, ed. *PLoS One*. 2025;20(6):e0325001. doi:10.1371/journal.pone.0325001.
50. Diaz-Torres IA, Cabrera-Takane ID, Ortega-Vargas FY, et al. Epidemiological and Clinical Changes in RSV-Associated Pneumonia in Children in Mexico Before and During the COVID 19 Pandemic. *Infectious Disease Reports*. 2025;17(6):139. doi:10.3390/idr17060139.
51. Di Maio VC, Scutari R, Mastropaolo M, et al. Viral Burden of Respiratory Syncytial Virus and Viral Coinfections as Factors Regulating Paediatric Disease Severity. *Viruses*. 2025;17(9):1236. doi:10.3390/v17091236.
52. Correia W, Dorta‐Guerra R, Sanches M, Valladares B, De Pina‐Araújo IIM, Carmelo E. Epidemiological and clinical profile of viral respiratory infections in children under 5 years at pre‐ and post‐ COVID ‐19 era in Praia, Cabo Verde. *Tropical Med Int Health*. 2025;30(7):694-703. doi:10.1111/tmi.14125.
53. Chen S, Xu X, Wu M, Zhou L, Wang Y. Risk factors for recurrent wheezing after infant bronchiolitis: a 6-year single-centre follow up study in China. *Front Pediatr*. 2025;13:1549475. doi:10.3389/fped.2025.1549475.
54. Chen P, Li Y, Li S, Hua X, Liu Y. Analysis of epidemic trend of respiratory pathogens in children after long-term pathogen isolation. *PeerJ*. 2025;13:e19710. doi:10.7717/peerj.19710.
55. Cetin M, Turan C, Yurumez C, Yurtseven A, Cicek C, Saz EU. Shifting trends in influenza and rising HBoV cases: a comparative analysis over 3-winter seasons. *BMC Infect Dis*. 2025;25(1):1617. doi:10.1186/s12879-025-12051-6.
56. Arimura K, Kan-o K, Sato Y, et al. Age-Dependent Risk of Bronchial Asthma Exacerbation in Respiratory Syncytial Virus Co-infection. *Lung*. 2025;203(1):91. doi:10.1007/s00408-025-00847-x.
57. Alamiri HA, Hamwi S, Alsamri MT, et al. Epidemiological characterization and seasonality of respiratory syncytial virus in the United Arab Emirates: A five-year study at a tertiary care hospital. *Journal of Infection and Public Health*. 2025;18(12):103007. doi:10.1016/j.jiph.2025.103007.
58. Alamiri HA, Hamwi S, Alsamri MT, et al. Epidemiological characterization and seasonality of respiratory syncytial virus in the United Arab Emirates: A five-year study at a tertiary care hospital. *Journal of Infection and Public Health*. 2025;18(12):103007. doi:10.1016/j.jiph.2025.103007.
59. Akhtar Z, Notaras A, Tawfiq E, et al. Epidemiology of respiratory syncytial virus within a New South Wales-based multi-centre health district between 2018 and 2024 in Australia. *Pathology*. Published online August 2025:S0031302525002661. doi:10.1016/j.pathol.2025.06.011.
60. Cai L, Gao XM, Zhu FC, et al. Multicenter analysis of common non-bacterial pathogens in children hospitalized with acute respiratory infections in four regions of Fujian Province, 2023. *Chin J Prev Med*. 2025;59(10):1665-1675. doi: 10.3760/cma.j.cn112150-20241211-00993.
61. Luo XJ, Wang W, Ren ZM, et al. Epidemiological characteristics of respiratory syncytial virus among inpatients in a children’s hospital in Shenzhen from 2020 to 2023. *Chin J Prev Med*. 2025;59(4):484-489. doi: 10.3760/cma.j.cn112150-20240912-00735.
62. Amarin JZ, Toepfer AP, Spieker AJ, et al. Respiratory Syncytial Virus Co-Detection With Other Respiratory Viruses Is Not Significantly Associated With Worse Clinical Outcomes Among Children Aged <2 Years: New Vaccine Surveillance Network, 2016-2020. *Clin Infect Dis*. Published online May 9, 2025. doi: 10.1093/cid/ciaf194.
